# Whole-Genome DNA Methylation Sequencing Reveals Epigenetic Changes in Myelodysplastic Syndromes

**DOI:** 10.3389/fonc.2022.897898

**Published:** 2022-06-29

**Authors:** Jing-dong Zhou, Zi-jun Xu, Ye Jin, Xin-long Zhang, Yu Gu, Ji-chun Ma, Xiang-mei Wen, Jiang Lin, Ting-juan Zhang, Jun Qian

**Affiliations:** ^1^ Department of Hematology, Affiliated People’s Hospital of Jiangsu University, Zhenjiang, China; ^2^ Zhenjiang Clinical Research Center of Hematology, Zhenjiang, China; ^3^ The Key Lab of Precision Diagnosis and Treatment in Hematologic Malignancies of Zhenjiang City, Zhenjiang, China; ^4^ Laboratory Center, Affiliated People’s Hospital of Jiangsu University, Zhenjiang, China; ^5^ Department of Hematology, The People’s Hospital of Danyang, Zhenjiang, China; ^6^ Department of Oncology, Affiliated People’s Hospital of Jiangsu University, Zhenjiang, China

**Keywords:** methylation, *LEP*, myelodysplastic syndromes, genome, epigenetics

## Abstract

Epigenetic dysregulation of cancer-associated genes has been identified to contribute to the pathogenesis of myelodysplastic syndromes (MDS). However, few studies have elucidated the whole-genome DNA methylation in the initiation pathogenesis of MDS. Reduced representation bisulfite sequencing was performed in five *de novo* MDS patients and four controls to investigate epigenetic alterations in MDS pathogenesis. The mean global methylation in five MDS patients showed no significant difference compared with the four controls. In depth, a total of 1,459 differentially methylated fragments, including 759 hypermethylated and 700 hypomethylated fragments, were identified between MDS patients and controls. Targeted bisulfite sequencing further identified that hypermethylation of *DLEU7*, *FOXR1*, *LEP*, and *PANX2* were frequent events in an additional cohort of MDS patients. Subsequently, *LEP* hypermethylation was confirmed by real-time quantitative methylation-specific PCR in an expanded cohort of larger MDS patients. In clinics, *LEP* hypermethylation tended to be associated with lower bone marrow blasts and was significantly correlated with *U2AF1* mutation. Survival analysis indicated that *LEP* hypermethylation was associated with a markedly longer survival time but was not an independent prognostic biomarker in MDS patients. Functional studies revealed pro-proliferative and anti-apoptotic effects of leptin in the MDS cell line SKM-1, and it was significantly associated with cell growth and death as well as the Toll-like receptor and NF-kappa B signaling pathways. Collectively, our findings demonstrated that whole-genome DNA methylation analysis identified novel epigenetic alterations such as *DLEU7*, *FOXR1*, *LEP*, and *PANX2* methylations as frequent events in MDS. Moreover, *LEP* might play a role in MDS pathogenesis, and *LEP* hypermethylation was associated with longer survival but not as an independent prognostic biomarker in MDS.

## Introduction

Myelodysplastic syndromes (MDSs) represent a group of diverse clonal hematopoietic disorders characterized by peripheral blood cytopenia, ineffective production of blood cells, and high risk of transformation to acute myeloid leukemia (AML) (1). Cytogenetic abnormalities and genetic mutations play crucial roles in the pathogenssis of MDS, and have been proved to be clearly associated with MDS diagnosis and prognosis (2). Moreover, epigenetic dysregulation of cancer-associated genes has been identified as contributing to the pathogenesis of MDS (3). In particular, hypermethylation of CpG islands (CGIs) located at the promoter region of tumor suppressor genes (TSGs) and their consequent gene silencing have been revealed in MDS (4). Recently, epigenetic alterations as new biological makers have also have been widely used for predicting prognosis and risk of AML in patients with MDS (5–7). Importantly, epigenetic therapies with demethylating agents such as azacytidine and decitabine have demonstrated clinical effectiveness and have been approved by the Food and Drug Administration (FDA) as antitumor agents for the treatment of MDS (8, 9).

Previously, we determined the genome-wide DNA methylation alterations during MDS progression by reduced representation bisulfite sequencing (RRBS) and revealed that genome-wide DNA hypermethylation changes were a common phenomenon during MDS progression (10). Moreover, hypermethylation of *ZNF300*, *DLX5*, *SOX30*, *ID4*, and *GPX3* genes was associated with the prognosis and disease progression of MDS (10–14). However, only a few studies have elucidated the whole-genome DNA methylation in the initiation pathway of MDS. Based on our previous study, we further re-analyzed the RRBS data of bone marrow (BM) samples from five *de novo* MDS patients and four controls to investigate epigenetic alterations in MDS pathogenesis.

## Methods

### Patients and Samples

In this study, three independent cohorts of MDS patients and controls were included after informed consent was obtained. Firstly, a total of five *de novo* MDS patients and four healthy donors from the Affiliated People’s Hospital of Jiangsu University and the First Affiliated Hospital of Soochow University were enrolled in the RRBS. Next, another cohort of 36 *de novo* MDS and 25 healthy donors treated at the Affiliated People’s Hospital of Jiangsu University was used in the targeted bisulfite sequencing. Lastly, the third cohort of 105 *de novo* MDS and 46 healthy donors treated at the Affiliated People’s Hospital of Jiangsu University was included in the real-time quantitative methylation-specific PCR (RQ-MSP) analysis. BM was collected from all MDS patients at the time of diagnosis with controls. BM mononuclear cells (BMMNCs) were separated by density-gradient centrifugation using Lymphocyte Separation Medium (Solarbio, Beijing, China). Subsequently, DNA extraction was carried out based on the instructions of the manufacturer (10). This study was approved by the Ethics Committee of Affiliated People’s Hospital of Jiangsu University.

### RRBS and Targeted Bisulfite Sequencing

RRBS and targeted bisulfite sequencing (MethylTarget) were performed by Genesky Biotechnologies Inc. (Shanghai, China). A detailed description of the RRBS and MethylTarget assay was described in our previous report (10). The primers of the selected genes used in MethylTarget are shown in **Table S1**.

### Bisulfite Modification and RQ-MSP

In our previous literature (10), bisulfite conversion of genomic DNA was reported. RQ-MSP was further performed to quickly detect the methylation level of *LEP* by using AceQ qPCR SYBR Green Master Mix (Vazyme Biotech Co., Piscataway, NJ) as reported (15). Detailed information regarding the PCR can be referred to in our previous literature (15).

### Cell Lines, Cell Culture, and Reagents

The MDS cell line SKM-1 was cultured in RPMI 1640 medium containing 10% fetal calf serum (ExCell Bio, Shanghai, China) and grown at 37°C in a 5% CO2 humidified atmosphere (10). Human recombinant human leptin (R&D Systems, Minneapolis, MN) was dissolved in the medium to a working concentration of 100 ng/ml.

### Cell Growth Assays

The tested cells were seeded in 96-well plates (at a density of 5 × 10^3^ cells/well) in triplicate and cultured for 0, 24, 48, and 72 h, respectively. A Cell Counting Kit-8 (Dojindo, Kumamoto, Japan) was added to each well and incubated for 2 h, and was measured using a microplate reader at the absorbance at 450 nm. The rate of cell growth was calculated by the OD value.

### Cell Apoptosis Assays

The tested cells were cultured with serum-free RPMI 1640 medium for 48 h in 6-well plates (at a density of 5 × 10^5^ cells/well) in triplicate. An Annexin V PE Apop Dtec Kit (BD Pharmingen, San Diego, CA) was used to analyze the apoptosis rate by flow cytometry according to the protocols of the manufacturer.

### RNA Sequencing

Next Generation Sequencing (NGS) RNA-Seq was performed to analyze the transcriptomes of the tested cells. Total RNA was isolated using the QIAamp RNA Blood Mini Kit (QIAGEN, Düsseldorf, Germany) according to the instructions of the manufacturer. RNA samples were analyzed by the BGISEQ-500 platform (BGI, Wuhan, China). The details of RNA-Seq could be referred to in previous reports (16).

### Public Datasets and Bioinformatics Analysis

A cohort of 159 MDS patients and 17 healthy controls downloaded from the Gene Expression Omnibus (GEO) database (GSE58831) (https://www.ncbi.nlm.nih.gov/geo/query/acc.cgi?acc=GSE58831) was used to identify the mRNA expression of *LEP* in MDS.

### Statistics

Statistical analysis was accomplished using the SPSS 22.0 and GraphPad Prism 5.0 software packages. Comparisons of continuous variables were done by the Independent T/Mann–Whitney’s U test, whereas comparisons of categorical variables were analyzed using Pearson Chi-square/Fisher exact tests. A Spearman correlation test was performed to analyze the correlation between the results obtained using RQ-MSP and targeted bisulfite sequencing in the detection of *LEP* methylation. Kaplan–Meier analysis (Log-rank test) and Cox regression analysis (univariate and multivariate proportional hazard regression) were performed to evaluate the prognostic impact of *LEP* methylation on leukemia-free survival (LFS) and overall survival (OS) of MDS patients. The statistical results were considered significantly different if two-sided *P-*values were less than 0.05.

## Results

### Genome-Wide Methylation Analysis in MDS Patients

To identify epigenetic alterations occurring in MDS, we performed RRBS in five newly diagnosed MDS patients and four healthy donors. The sequencing data of four newly diagnosed MDS patients and four healthy donors were submitted to the NCBI SRA databases (PRJNA670308) previously. Sequencing data of one remaining MDS patients are available from the corresponding author on reasonable request. The details of the sequencing data were described in our previous study (10). However, the mean global methylation in five *de novo* MDS patients (51.2, 38.9, 43.3, 42.3, and 44.8%) showed no significant difference compared with four controls (51.0, 49.3, 49.4, and 45.4%) (*P* = 0.106, **Figure S1**).

**Figure 1:**
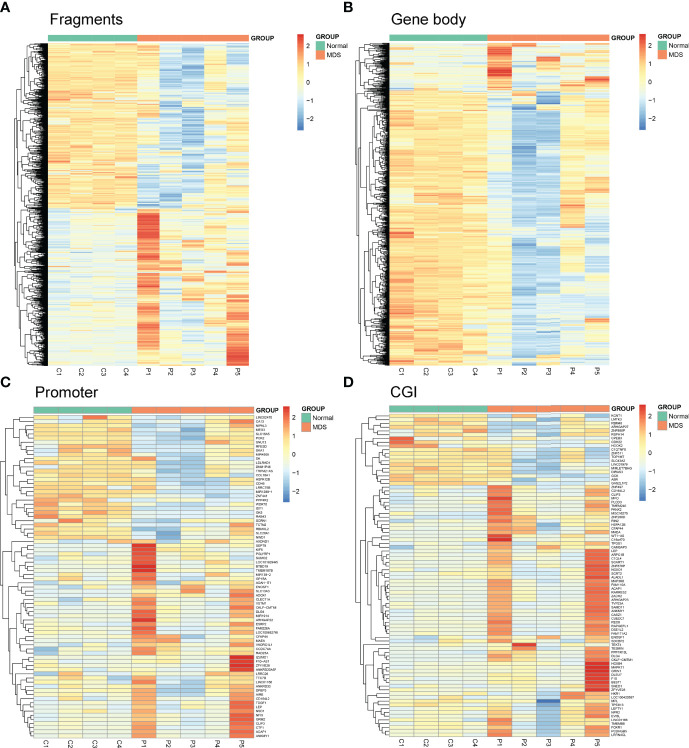
Heatmaps summarizing differentially methylated fragments/genes analyzed by the unit of *Mspl* fragments, CpG islands, gene body, and promoter. **(A)** Fragments; **(B)** Gene Body; **(C)** Promoter; **(D)** CGI. The fragments/genes that passed statistical significance (*P <*0.05, *Q <*0.05 and also had > 25% mean methylation difference) were considered as differentially methylated fragments/genes.

Next, we used *Mspl* fragments (40–220 bp) rather than individual CpG sites or a tiled window approach as the basic analysis units as in our previous report (10). The fragments that were statistically significant (*P <*0.05, *Q <*0.05, and also had >25% mean methylation difference) were considered differentially methylated fragments (DMFs). A total of 1459 DMFs, including 759 hypermethylated and 700 hypomethylated fragments, were identified between MDS patients and controls (**Figure 1A** and **Supplementary Table S2**). Moreover, both CpG islands (CGI), promoter ( ± 2,000 bp from transcription start site), and gene body were also used as the units of analysis, respectively. A total of 922 differentially methylated gene bodies (146 hypermethylated and 776 hypomethylated), 78 differentially methylated promoters (48 hypermethylated and 30 hypomethylated), and 87 differentially methylated CGI (65 hypermethylated and 22 hypomethylated) were identified between MDS patients and controls (**Figures 1B–D** and **Supplementary Table S2**).

**Figure 2:**
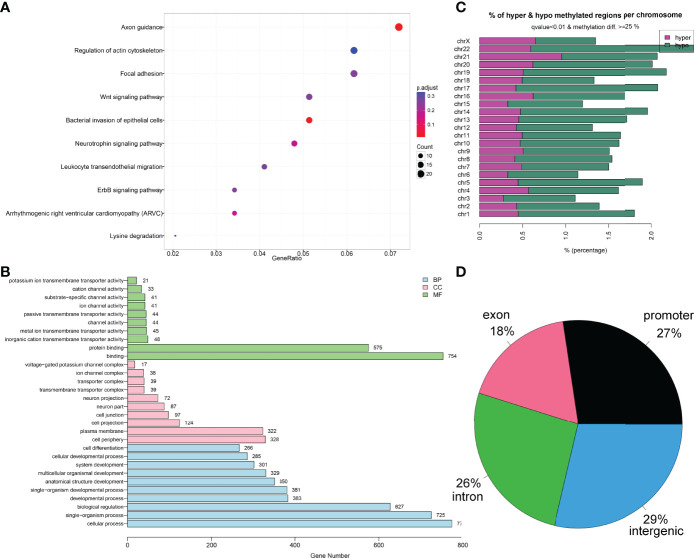
Bioinformatics analysis of the differentially methylated fragments/genes in MDS. **(A)** Kyoto Encyclopedia of Genes and Genomes analysis of 1,459 differentially methylated fragments/genes between MDS patients and controls. **(B)** Gene Ontology analysis of 1,459 differentially methylated fragments/genes between MDS patients and controls. **(C, D)** The locations of 1,459 differentially methylated fragments in the distribution of chromosome and gene region.

Finally, the Gene Ontology (GO) and Kyoto Encyclopedia of Genes and Genomes (KEGG) enrichment analysis of the 1,459 DMFs-related genes are shown in **Figures 2A, B**. Moreover, the locations of 1,459 DMFs in the distribution of chromosome and gene region are presented in **Figures 2C, D**.

### Identification and Validation of Candidate DMFs in MDS Patients

**Figure 3:**
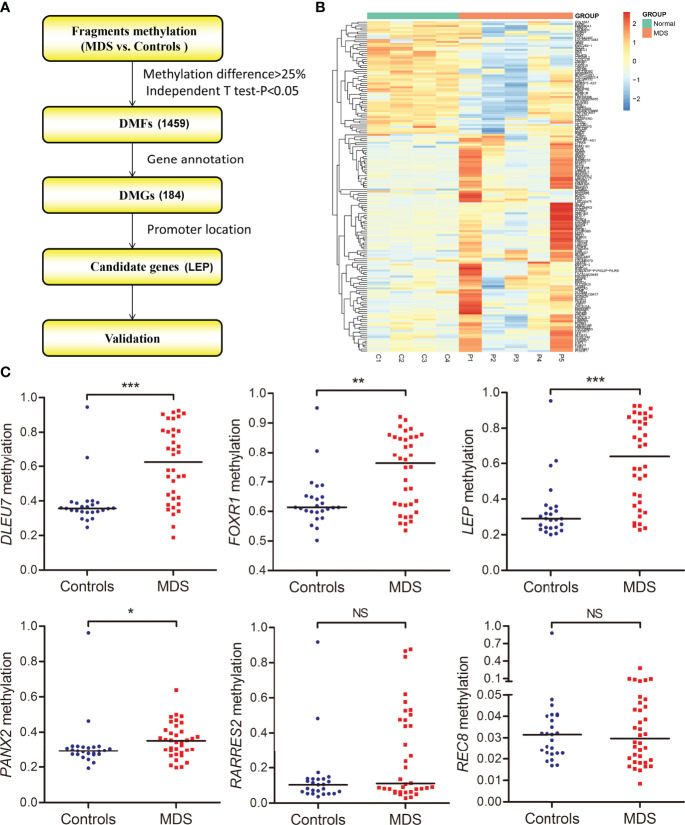
Identification and validation of differentially methylated genes in MDS. **(A)** The flowchart of the differentially methylated genes screening. The fragments that passed statistical significance (*P <*0.05, *Q <*0.05 and also had > 25% mean methylation difference) were considered as differentially methylated fragments/genes. **(B)** Heatmaps summarizing differentially methylated fragments/genes in MDS. **(C)** The methylation level of the candidate genes in additional samples of *de novo* MDS (n = 36) and controls (n = 25) analyzed by targeted bisulfite sequencing. *P*-values were calculated using the Mann–Whitney U-test. NS, no significance; *P <0.05; **P <0.01; ***P <0.001.

As is well known, the promoter CpG site hypermethylation is associated with gene silencing, and plays a crucial role in cancer development. Here, to further identify the candidate genes involved in MDS, we first annotated 1,459 DMFs as differentially methylated genes (DMGs), and then selected the promoter-associated DMGs, and finally selected the candidate genes for validation (**Figure 3A**). Following the procedure, we obtained 184 DMGs (128 hypermethylated genes and 56 hypomethylated genes), which may play a crucial role in MDS pathogenesis (**Figure 3B**).

The targeted bisulfite sequencing methodology MethylTarget (GENESKY, Shanghai, China) was performed in an additional cohort of 36 MDS and 25 controls to further validate the six candidate genes (*DLEU7*, *FOXR1*, *LEP*, *PANX2*, *RARRES2*, and *REC8*), which may have potential biological functions in cancers predicted by Coremine analysis (http://www.coremine.com/medical/#search). Besides *RARRES2* and *REC8*, the methylation level of *DLEU7*, *FOXR1*, *LEP*, and *PANX2* in MDS patients was markedly increased compared with controls (**Figure 3**).

### Further Confirmation of LEP Methylation in a Larger Cohort of MDS Patients

**Figure 4:**
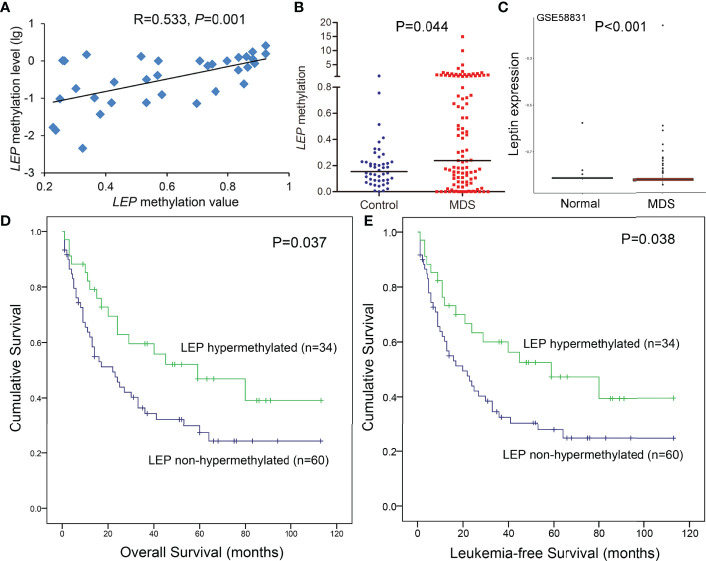
Further confirmation of *LEP* methylation in MDS patients together with its prognostic value. **(A)** The correlation of *LEP* methylation results between the targeted bisulfite sequencing and RQ-MSP. The correlation was analyzed by Spearman correlation test. **(B)** The methylation level of the *LEP* in larger samples of controls (n = 46) and *de novo* MDS (n = 105) and AML patients (n = 170) analyzed by RQ-MSP. P-values were calculated using the Mann–Whitney U-test. **(C)** Leptin expression in MDS patients from public GEO datasets. **(D, E)** The impact of *LEP* methylation on overall survival and leukemia-free survival of MDS patients. Survival was analyzed though Kaplan–Meier analysis using Log-rank test.

Hypermethylation of *LEP* was further confirmed in a larger cohort of 105 MDS patients and 46 controls by RQ-MSP developed previously (15). The results obtained by RQ-MSP and targeted bisulfite sequencing in the detection of *LEP* methylation among MDS patients were highly correlated with each other (R = 0.533, *P* = 0.001, **Figure 4A**). Moreover, the *LEP* methylation level in MDS patients was markedly higher than that in controls, as expected (*P* = 0.044, **Figure 4B**). Because of the limited mRNA samples in our MDS cohort, we used the public GEO data to identify the expression of *LEP* in MDS patients. As shown in **Figure 4C**, *LEP* mRNA expression was significantly reduced in MDS patients compared with normal controls (*P <*0.001).

To analyze the clinical relevance of *LEP* methylation in MDS, we divided the patients into two groups (*LEP* hypermethylated and non-hypermethylated) based on the cutoff value of 0.569 (set as “mean + 2SD” in controls). As shown in **Table 1**, there were no marked differences when comparing the two groups with regard to sex, age, white blood cells, hemoglobin, platelets, and WHO and IPSS classifications (all *P >*0.05, **Table 1**). However, *LEP* hypermethylation tended to be associated with lower BM blasts (*P* = 0.052, **Table 1**) and was significantly correlated with *U2AF1* mutation (*P* = 0.016, **Table 1**).

**Table 1:** Comparison of clinical and laboratory features between *LEP* hypermethylated and non-hypermethylated MDS patients.

Patient’s features	Non-hypermethylated (n = 68)	Hypermethylated (n = 37)	*P*-value
Sex (male/female)	39/29	19/18	0.682
Median age, years (range)	62.5 (14–86)	52 (20–86)	0.123
Median WBC, ×10^9^/L (range)	2.7 (0.7–26.6)	2.85 (0.6–82.4)	0.706
Median hemoglobin, g/L (range)	65.5 (29–140)	59 (26–118)	0.149
Median platelets, ×10^9^/L (range)	65 (1–1,176)	43.5 (0–505)	0.110
Median BM blasts, % (range)	6 (0–18)	2 (0–19)	0.052
WHO classifications			0.788
RCUD/RARS	9	5	
RCMD/RCMD-RS	24	16	
RAEB-1	11	8	
RAEB-2	21	7	
MDS with isolated del(5q)	2	1	
MDS-U	1	0	
IPSS scores			0.294
Low	7	6	
Int-1	33	21	
Int-2	13	8	
High	8	1	
No data	7	1	
Gene mutations			
*CEBPA* (+/−)	1/61	2/30	0.266
*IDH1/2* (+/−)	3/59	1/31	1.000
*DNMT3A* (+/−)	2/60	1/31	1.000
*U2AF1* (+/−)	1/61	5/27	0.016
*SRSF2* (+/−)	2/60	1/31	1.000
*SF3B1* (+/−)	3/59	3/29	0.406
*SETBP1* (+/−)	0/62	1/31	0.340

MDS, myelodysplastic syndromes; WBC, white blood cells; BM, bone marrow; WHO, World Health Organization; IPSS, International Prognostic Scoring System.

### Prognostic Effect of LEP Methylation in MDS Patients

The prognostic effect of *LEP* methylation on OS and LFS was further analyzed in MDS patients. Interestingly, Kaplan–Meier analysis demonstrated that patients with *LEP* hypermethylation exhibited markedly longer OS and LFS times than patients with *LEP* non-hypermethylation (*P* = 0.037 and 0.038, respectively, **Figures 4D, E**). However, *LEP* hypermethylation was not a prognostic biomarker independently affecting OS and LFS in MDS patients (*P* = 0.540, **Table 2**) by Cox regression analysis.

**Table 2:** Cox regression analyses of variables for overall survival in MDS patients.

Variables	Univariate analyses		Multivariate analyses	
	Hazard ratio (95% CI)	*P*-value	Hazard ratio (95% CI)	*P*-value
*LEP* methylation	0.554 (0.314–0.978)	0.042	0.812 (0.417–1.581)	0.540
Age	2.913 (1.668–5.088)	0.000	3.271 (1.709–6.259)	0.000
IPSS risks	1.793 (1.022–3.145)	0.042	1.959 (1.048–3.661)	0.035
*CEBPA* mutation	0.429 (0.059–3.115)	0.403		
*IDH1/2* mutation	0.969 (0.301–3.117)	0.958		
*U2AF1* mutation	0.850 (0.305–2.368)	0.756		
*SF3B1* mutation	1.570 (0.487–5.062)	0.450		
*SRSF2* mutations	3.702 (1.124–12.188)	0.031	3.455 (0.998–11.964)	0.050
*SETBP1* mutations	9.798 (1.225–78.359)	0.031	37.817 (4.183–341.907)	0.001
*DNMT3A* mutation	3.241 (0.986–10.654)	0.053	3.123 (0.895–10.894)	0.074

IPSS, International Prognostic Scoring System. Variables including age (≤60 vs. >60 years old), IPSS scores (Low vs. Int-1 vs. Int-2 vs. High), LEP methylation (non-hypermethylated vs. hypermethylated), and gene mutations (mutant vs. wild-type). Multivariate analysis includes variables with P <0.200 in univariate analysis.

### Biological Functions of LEP in MDS Cells

**Figure 5:**
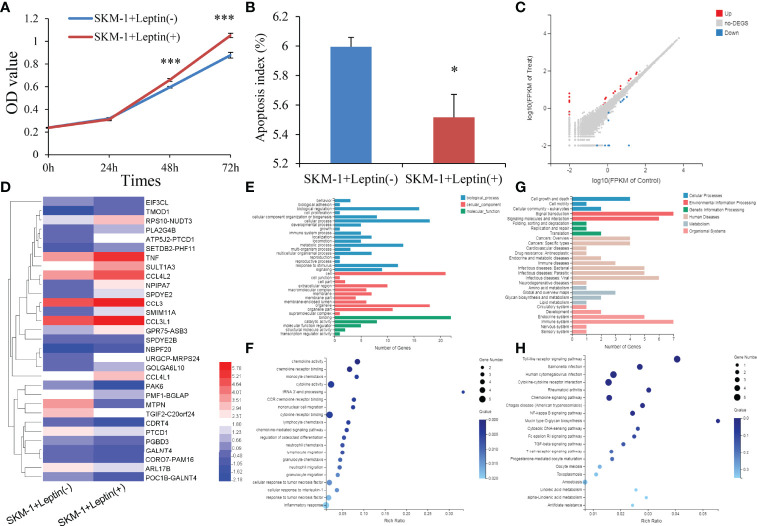
Biological functions of *LEP* in MDS cell line SKM-1. **(A)** The proliferation ability in SKM-1 cells before and after human recombinant leptin treatment. **(B)** The apoptosis ability in SKM-1 cells before and after human recombinant leptin treatment. **(C)** Volcano plot of differentially expressed genes between SKM-1 cells before and after human recombinant leptin treatment. **(D)** Expression heatmap of differentially expressed genes between SKM-1 cells before and after human recombinant leptin treatment. **(E, F)** Gene Ontology analysis of differentially expressed genes. **(G, H)** Gene Ontology and Kyoto Encyclopedia of Genes and Genomes analysis of differentially expressed genes. *P*-values were calculated using the independent T-test. *P < 0.05; ***P < 0.001.

To determine the potential role of *LEP* during MDS pathogenesis, we carried out gain-of-function experiments in the MDS cell line SKM-1 *in vitro*. Interestingly, SKM-1 cells treated with human recombinant leptin exhibited a higher growth rate and a lower apoptosis rate than those treated without human recombinant leptin (**Figures 5A,B**). The biological functions of leptin seemed to be contrary to the hypermethylation pattern in MDS.

We next performed mRNA-sequencing of SKM-1 cells before and after treating with human recombinant leptin to get a better understanding of the biological network in MDS affected by aberrant leptin expression. A total of 31 differential expressed genes (DEGs) including 20 upregulated and 11 downregulated were identified between two groups (|log2 FC| >1, FDR <0.05 and *P <*0.05) (**Figures 5C, D**). Moreover, the analysis of Gene Ontology (GO) and Kyoto Encyclopedia of Genes and Genomes (KEGG) enrichment revealed that these genes were involved in cell growth and death (**Figures 5E, F**) as well as Toll-like receptor and NF-kappa B signaling pathways (**Figures 5G, H**).

## Discussion

Epigenetic alterations, especially aberrant DNA methylations, play a vital rols in MDS progression. Previously, Jiang et al. reported that aberrant DNA methylation was a dominant mechanism in MDS progression to AML (17). Similarly, our previous study also confirmed that whole-genome DNA hypermethylation was a frequent event during MDS progression (10). Interestingly, our study did not observe genome-wide DNA hypermethylation in the MDS stage compared with controls, while the whole-genome methylation level in MDS seems to be lower than in controls. Deep analysis of differentially methylated CpG sites revealed that the gene body showed more hypomethylated changes than hypermethylation alterations, whereas CpG islands and promoters showed the opposite phenomena. As is well known, promoter CpG island hypermethylation is associated with TSG silencing during oncogenesis. Gene body hypomethylation harbor extensive intragenic transcriptional activity and is prone to cancer-associated dysregulation (18).

In this study, we further identified several DNA methylation-associated genes as novel epigenetic alterations in MDS. Although aberrant methylation of these candidate genes, including *FOXO3* and *TERT*, has previously been reported in MDS (19, 20), most others remain to be elucidated. Interestingly, some of these genes, such as *LEP*, *ALOX12*, *RARRES2*, *DLEU7*, and *FOXR1*, have been reported to be associated with other hematologic malignancies, including AML, chronic lymphocytic leukemia (CLL), and B-cell lymphoma (15, 21–24). Moreover, methylations in *RARRES2*, *REC8*, *WNK2*, and *PANX2* have been demonstrated with potential roles in solid cancers (25–28). Therefore, we focused on these genes which were clearly associated with other hematologic malignancies or solid tumors for further validation and revealed that methylations of *DLEU7*, *FOXR1*, *LEP*, and *PANX2* were frequent events in MDS. However, due to the limited samples detected in targeted bisulfite sequencing, further investigations are needed to confirm our results.

Since our research group has determined the obesity-related gene *LEP* methylation in AML, the *LEP* methylation pattern in MDS patients has come to our attention. Based on our previous study, *LEP* hypermethylation was not present during MDS progression (10). Moreover, hypermethylation of *LEP* was a frequent event in primary AML and MDS-derived AML, but they showed no significant difference between primary AML and MDS-derived AML (15). In this study, we further confirmed the *LEP* hypermethylation phenomenon in a larger cohort of MDS patients, but it was not an independent prognostic biomarker affecting LFS in MDS. These results indicated that *LEP* methylation may only play a vital role in the initiation pathogenesis of MDS rather than disease progression.

As is well known, promoter CpG island hypermethylation is associated with gene silencing. It is a pity that we did not evaluate *LEP* methylation with *LEP* expression in MDS, which was mainly caused by the limited samples. Nevertheless, our previous study has proved the significantly negative association between *LEP* methylation and *LEP* expression in AML (15). Moreover, we further identified the reduced expression of *LEP* in MDS patients by using public GEO datasets. Although methylation of *LEP* and its direct role of leptin protein were less reported in MDS, leptin expression in MDS has been increasingly investigated. Dalamaga et al. revealed that low leptin concentrations were observed in low-risk MDS patients with normal or good prognostic karyotype (29). Subsequently, the same research group further reported that free leptin was associated with a higher risk of MDS, particularly among overweight/obese individuals (30). In our study, although the significant association of *LEP* hypermethylation with IPSS risks was not observed, we showed that *LEP* hypermethylation, as a frequent event in MDS, was associated with longer OS and LFS. Similarly, Kraakman et al. demonstrated that leptin-deficient obesity prolongs survival in a murine model of MDS (31). The functional studies showed pro-proliferative and anti-apoptotic effects of leptin in MDS in accordance with a previous study (32). These “conflicting” results might suggest that *LEP* hypermethylation-mediated leptin expression was an early event in MDS initiation but did not act as a tumor suppressor, and that they played a protective role during MDS progression, leading to favorable prognosis. These findings provided a theoretical basis and opened new insights for developing leptin-related targeted therapy in MDS.

Collectively, our findings indicated that whole-genome DNA methylation analysis identified novel epigenetic alterations such as *DLEU7*, *FOXR1*, *LEP*, and *PANX2* methylations as frequent events in MDS. Moreover, *LEP* might play a role in MDS pathogenesis, and *LEP* hypermethylation was associated with longer survival but not as an independent prognostic biomarker in MDS.

## Data Availability Statement

The datasets presented in this study can be found in online repositories. The names of the repository/repositories and accession number(s) can be found below: https://www.ncbi.nlm.nih.gov/bioproject/PRJNA670308.

## Ethics Statement

The studies involving human participants were reviewed and approved by Ethics Committee of the Affiliated People’s Hospital of Jiangsu University. The patients/participants provided their written informed consent to participate in this study.

## Author Contributions

J-dZ and JQ conceived and designed the experiments. T-jZ and J-dZ performed the experiments. Z-jX analyzed the data and provided bioinformatics analysis. YG and X-lZ collected the clinical data. J-cM, X-mW and JL provided the technical and financial supports. J-dZ wrote the paper. All authors listed have made a substantial, direct, and intellectual contribution to the work and approved it for publication.

## Funding

The work was supported by the National Natural Science Foundation of China (81900166, 81900163, 81970118), the Zhenjiang Clinical Research Center of Hematology (SS2018009), the Social Development Foundation of Zhenjiang (SH2020055, SH2021052), the Medical Field of Zhenjiang “Jin Shan Ying Cai” Project, Medical Education Collaborative Innovation Fund of Jiangsu University (JDY2022011), and the Scientific Research Foundation of Affiliated People’s Hospital of Jiangsu University for PhD (KFB202002, KFB202202).

## Conflict of Interest

The authors declare that the research was conducted in the absence of any commercial or financial relationships that could be construed as a potential conflict of interest.

## Publisher’s Note

All claims expressed in this article are solely those of the authors and do not necessarily represent those of their affiliated organizations, or those of the publisher, the editors and the reviewers. Any product that may be evaluated in this article, or claim that may be made by its manufacturer, is not guaranteed or endorsed by the publisher.

## Abbreviations

MDS, myelodysplastic syndromes; AML, acute myeloid leukemia; CGI, CpG islands; TSGs, tumor suppressor genes; FDA, Food and Drug Administration; RRBS, reduced representation bisulfite sequencing; BM, bone marrow; BMMNCs, BM mononuclear cells; RQ-MSP, real-time quantitative methylation-specific PCR; NGS, Next Generation Sequencing; GEO, Gene Expression Omnibus; LFS, leukemia-free survival; OS, overall survival; GO, Gene Ontology; KEGG, Kyoto Encyclopedia of Genes and Genomes; DMFs, differentially methylated fragments; DMGs, differentially methylated genes; DEGs, differential expressed genes; CLL, chronic lymphocytic leukemia.

## References

[1] Cazzola M. Myelodysplastic Syndromes. N Engl J Med (2020) 383(14):1358–74. doi: 10.1056/NEJMra1904794 32997910

[2] Garcia-Manero G Chien KS Montalban-Bravo G. Myelodysplastic Syndromes: 2021 Update on Diagnosis, Risk Stratification and Management. Am J Hematol (2020) 95(11):1399–420. doi: 10.1002/ajh.25950 32744763

[3] Heuser M Yun H Thol F. Epigenetics in Myelodysplastic Syndromes. Semin Cancer Biol (2018) 51:170–9. doi: 10.1016/j.semcancer.2017.07.009 PMC7116652 28778402

[4] Ye F Li N. Role of P15(INK4B) Methylation in Patients With Myelodysplastic Syndromes: A Systematic Meta-Analysis. Clin Lymphoma Myeloma Leuk (2019) 19(6):e259–65. doi: 10.1016/j.clml.2019.03.013 31023595

[5] Cabezón M Malinverni R Bargay J Xicoy B Marcé S Garrido A. Different Methylation Signatures at Diagnosis in Patients With High-Risk Myelodysplastic Syndromes and Secondary Acute Myeloid Leukemia Predict Azacitidine Response and Longer Survival. Clin Epigenetics (2021) 13(1):9. doi: 10.1186/s13148-021-01002-y 33446256 PMC7809812

[6] Bond DR Lee HJ Enjeti AK. Unravelling the Epigenome of Myelodysplastic Syndrome: Diagnosis, Prognosis, and Response to Therapy. Cancers (2020) 12(11):3128. doi: 10.3390/cancers12113128 PMC7692163 33114584

[7] Shen L Kantarjian H Guo Y Lin E Shan J Huang X. DNA Methylation Predicts Survival and Response to Therapy in Patients With Myelodysplastic Syndromes. J Clin Oncol (2010) 28(4):605–13. doi: 10.1200/JCO.2009.23.4781 PMC2815995 20038729

[8] Quintás-Cardama A Santos FP Garcia-Manero G. Therapy With Azanucleosides for Myelodysplastic Syndromes. Nat Rev Clin Oncol (2010) 7(8):433–44. doi: 10.1038/nrclinonc.2010.87 20551943

[9] Estey EH. Epigenetics in Clinical Practice: The Examples of Azacitidine and Decitabine in Myelodysplasia and Acute Myeloid Leukemia. Leukemia (2013) 27(9):1803–12. doi: 10.1038/leu.2013.173 23757301

[10] Zhou JD Zhang TJ Xu ZJ Deng ZQ Gu Y Ma JC. Genome-Wide Methylation Sequencing Identifies Progression-Related Epigenetic Drivers in Myelodysplastic Syndromes. Cell Death Dis (2020) 11(11):997. doi: 10.1038/s41419-020-03213-2 33219204 PMC7679421

[11] Zhang TJ Xu ZJ Gu Y Wen XM Ma JC Zhang W. Identification and Validation of Prognosis-Related DLX5 Methylation as an Epigenetic Driver in Myeloid Neoplasms. Clin Transl Med (2020) 10(2):e29. doi: 10.1002/ctm2.29 32508046 PMC7403826

[12] Zhou JD Wang YX Zhang TJ Li XX Gu Y Zhang W. Identification and Validation of SRY-Box Containing Gene Family Member SOX30 Methylation as a Prognostic and Predictive Biomarker in Myeloid Malignancies. Clin Epigenetics (2018) 10:92. doi: 10.1186/s13148-018-0523-y 30002740 PMC6034269

[13] Zhou JD Zhang TJ Li XX Ma JC Guo H Wen XM. Epigenetic Dysregulation of ID4 Predicts Disease Progression and Treatment Outcome in Myeloid Malignancies. J Cell Mol Med (2017) 21(8):1468–81. doi: 10.1111/jcmm.13073 PMC5542913 28452111

[14] Zhou JD Lin J Zhang TJ Ma JC Yang L Wen XM. GPX3 Methylation in Bone Marrow Predicts Adverse Prognosis and Leukemia Transformation in Myelodysplastic Syndrome. Cancer Med (2017) 6(1):267–74. doi: 10.1002/cam4.984 PMC5269561 27891827

[15] Zhang TJ Xu ZJ Gu Y Ma JC Wen XM Zhang W. Identification and Validation of Obesity-Related Gene LEP Methylation as a Prognostic Indicator in Patients With Acute Myeloid Leukemia. Clin Epigenetics (2021) 13(1):16. doi: 10.1186/s13148-021-01013-9 33485366 PMC7824952

[16] Gorenjak M Zupin M Jezernik G Skok P Potočnik U. Omics Data Integration Identifies ELOVL7 and MMD Gene Regions as Novel Loci for Adalimumab Response in Patients With Crohn's Disease. Sci Rep (2021) 11(1):5449. doi: 10.1038/s41598-021-84909-z 33750834 PMC7970911

[17] Jiang Y Dunbar A Gondek LP Mohan S Rataul M O'Keefe C. Aberrant DNA Methylation is a Dominant Mechanism in MDS Progression to AML. Blood (2009) 113(6):1315–25. doi: 10.1182/blood-2008-06-163246 PMC2637194 18832655

[18] Mendizabal I Zeng J Keller TE Yi SV. Body-Hypomethylated Human Genes Harbor Extensive Intragenic Transcriptional Activity and are Prone to Cancer-Associated Dysregulation. Nucleic Acids Res (2017) 45(8):4390–400. doi: 10.1093/nar/gkx020 PMC5416765 28115635

[19] Sharifi MJ Zaker F Nasiri N Yaghmaie M. Epigenetic Changes in FOXO3 and CHEK2 Genes and Their Correlation With Clinicopathological Findings in Myelodysplastic Syndromes. Hematol Oncol Stem Cell Ther (2020) 13(4):214–9. doi: 10.1016/j.hemonc.2019.11.004 32217071

[20] Zhao X Tian X Kajigaya S Cantilena CR Strickland S Savani BN. Epigenetic Landscape of the TERT Promoter: A Potential Biomarker for High Risk AML/MDS. Br J Haematol (2016) 175(3):427–39. doi: 10.1111/bjh.14244 PMC5245983 27433923

[21] Ohgami RS Ma L Ren L Weinberg OK Seetharam M Gotlib JR. DNA Methylation Analysis of ALOX12 and GSTM1 in Acute Myeloid Leukaemia Identifies Prognostically Significant Groups. Br J Haematol (2012) 159(2):182–90. doi: 10.1111/bjh.12029 22924777

[22] Zhang J Zhou J Tang X Zhou LY Zhai LL Vanessa ME. Reduced Expression of Chemerin is Associated With Poor Clinical Outcome in Acute Myeloid Leukemia. Oncotarget (2017) 8(54):92536–44. doi: 10.18632/oncotarget.21440 PMC5696201 29190935

[23] Hammarsund M Corcoran MM Wilson W Zhu C Einhorn S Sangfelt O. Characterization of a Novel B-CLL Candidate Gene–DLEU7–located in the 13q14 Tumor Suppressor Locus. FEBS Lett (2004) 556(1-3):75–80. doi: 10.1016/S0014-5793(03)01371-1 14706829

[24] Pommerenke C Hauer V Zaborski M MacLeod RA Nagel S Amini RM. Chromosome 11q23 Aberrations Activating FOXR1 in B-Cell Lymphoma. Blood Cancer J (2016) 6(6):e433. doi: 10.1038/bcj.2016.43 27284737 PMC5141358

[25] Alholle A Brini AT Gharanei S Vaiyapuri S Arrigoni E Dallol A. Functional Epigenetic Approach Identifies Frequently Methylated Genes in Ewing Sarcoma. Epigenetics (2013) 8(11):1198–204. doi: 10.4161/epi.26266 24005033

[26] Yu J Liang Q Wang J Wang K Gao J Zhang J. REC8 Functions as a Tumor Suppressor and is Epigenetically Downregulated in Gastric Cancer, Especially in EBV-Positive Subtype. Oncogene (2017) 36(2):182–93. doi: 10.1038/onc.2016.187 PMC5241426 27212034

[27] Dutruel C Bergmann F Rooman I Zucknick M Weichenhan D Geiselhart L. Early Epigenetic Downregulation of WNK2 Kinase During Pancreatic Ductal Adenocarcinoma Development. Oncogene (2014) 33(26):3401–10. doi: 10.1038/onc.2013.312 23912455

[28] Xie CR Sun H Wang FQ Li Z Yin YR Fang QL. Integrated Analysis of Gene Expression and DNA Methylation Changes Induced by Hepatocyte Growth Factor in Human Hepatocytes. Mol Med Rep (2015) 12(3):4250–8. doi: 10.3892/mmr.2015.3974 PMC4526041 26099202

[29] Dalamaga M Nikolaidou A Karmaniolas K Hsi A Chamberland J Dionyssiou-Asteriou A. Circulating Adiponectin and Leptin in Relation to Myelodysplastic Syndrome: A Case-Control Study. Oncology (2007) 73(1-2):26–32. doi: 10.1159/000120995 18337619

[30] Dalamaga M Karmaniolas K Chamberland J Nikolaidou A Lekka A Dionyssiou-Asteriou A. Higher Fetuin-A, Lower Adiponectin and Free Leptin Levels Mediate Effects of Excess Body Weight on Insulin Resistance and Risk for Myelodysplastic Syndrome. Metabolism (2013) 62(12):1830–9. doi: 10.1016/j.metabol.2013.09.007 24140093

[31] Kraakman MJ Kammoun HL Dragoljevic D Al-Sharea A Lee MKS Flynn MC. Leptin-Deficient Obesity Prolongs Survival in a Murine Model of Myelodysplastic Syndrome. Haematologica (2018) 103(4):597–606. doi: 10.3324/haematol.2017.181958 29371326 PMC5865427

[32] Konopleva M Mikhail A Estrov Z Zhao S Harris D Sanchez-Williams G. Expression and Function of Leptin Receptor Isoforms in Myeloid Leukemia and Myelodysplastic Syndromes: Proliferative and Anti-Apoptotic Activities. Blood (1999) 93(5):1668–76. doi: 10.1182/blood.V93.5.1668.405a15_1668_1676 10029596

