# Supplementary material for: Whole-Genome DNA Methylation Sequencing Reveals Epigenetic Changes in Myelodysplastic Syndromes
Source: Front Oncol. 2022 Jun 29;12:897898. doi: 10.3389/fonc.2022.897898 (PMC9277050; doi:10.3389/fonc.2022.897898)

**Figure S1. Whole-genome methylation profiling in MDS patients.** The percentage of methylated CpG sites in 5 *de novo* MDS patients and 4 controls were demonstrated. P-values were calculated using the Independent T-test.


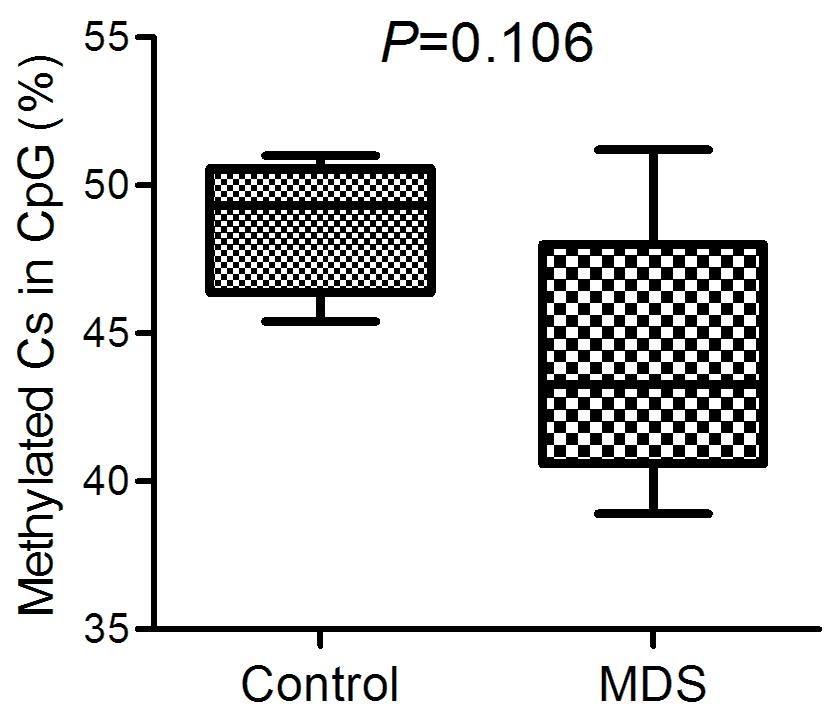

Supplement: Supplementary file 1 [file DataSheet_1.docx]
