# Supplementary material for: Whole-Genome DNA Methylation Sequencing Reveals Epigenetic Changes in Myelodysplastic Syndromes
Source: Front Oncol. 2022 Jun 29;12:897898. doi: 10.3389/fonc.2022.897898 (PMC9277050; doi:10.3389/fonc.2022.897898)
Supplement: Supplementary file 2 [file Table_1.docx]

**Supplementary Table S1. Primers used for MethylTarget sequencing, RQ-PCR, and RQ-MSP.**

| Primers | Primer sequence (5’to 3’) |
| --- | --- |
| MethylTarget sequencing |  |
| *DLEU7-*F | TTTGGTGGTTGATGGAGGTT |
| *DLEU7*-R | CTATAAAACCCAAAAACCRAAAAAC |
| *FOXR1*-F | GAAATAAGGGGTAAGGGGTTTG |
| *FOXR1*-R | CCTCACAAACRCCTTCTCAAATAC |
| *LEP*-F | GTGGGGTTTTGTGGTTTGTT |
| *LEP*-R | ACACACTACRAACCCAAAACTAACA |
| *PANX2*-F | TTTTTGTAGTTGAAGTTGGAGTTGT |
| *PANX2*-R | CACCTACRAACRCCCCCTCT |
| *RARRES2*-F | GTYGGGGGAAGGGGTAGG |
| *RARRES2*-R | CCCCATTCCTAACRACTAAAACTAAC |
| *REC8*-F | TTTTTATGATTGGTTTGTTGGTTGTT |
| *REC8*-R | CAAACCCCTAAACCTTACACTAACT |
|  |  |
| RQ-PCR |  |
| *LEP-*F | TTGGCCCTATCTTTTCTATG |
| *LEP*-R | GCATACTGGTGAGGATCTGT |
|  |  |
| RQ-MSP |  |
| *LEP*-MF | TCGGGGTTTTATTTTGTAATC |
| *LEP*-MR | CACGTCGCTACCCTAAAA |
| *LEP*-UF | GTTGGGGTTTTATTTTGTAATT |
| *LEP*-UR | ACACATCACTACCCTAAAA |

RQ-PCR: real-time quantitative PCR; RQ-MSP: real-time quantitative methylation-specific PCR
